# Effects of Extraction Conditions on Banana Peel Polyphenol Oxidase Activity and Insights into Inactivation Kinetics Using Thermal and Cold Plasma Treatment

**DOI:** 10.3390/foods10051022

**Published:** 2021-05-08

**Authors:** Daria Wohlt, Elena Schwarz, Andreas Schieber, Stephanie Bader-Mittermaier

**Affiliations:** 1 Department of Food Process Development, Fraunhofer Institute for Process Engineering and Packaging IVV, 85354 Freising, Germany; elenaschwarz@icloud.com (E.S.); stephanie.mittermaier@ivv.fraunhofer.de (S.B.-M.); 2 Institute of Nutritional and Food Sciences, Molecular Food Technology, University of Bonn, 53115 Bonn, Germany; schieber@uni-bonn.de

**Keywords:** enzyme characterization, *Musa acuminata* x *M. balbisiana*, *o*-diphenoloxidase, cold atmospheric pressure plasma, membrane-bound PPO, soluble PPO

## Abstract

The objective of this work was to characterize banana peel polyphenol oxidase (PPO) and to study the inactivation kinetics during thermal and cold atmospheric pressure plasma treatment. Since varietal differences in enzyme characteristics are a well-known phenomenon, ‘Prata’ banana peel PPO was characterized, and PPO activity and thermal stability of the peel PPO of the two dessert banana cultivars ‘Cavendish’ and ‘Prata’ were compared to identify the cultivar better suited for industrial food applications. A crude extract obtained from the peels of the Brazilian banana variety ‘Prata’ revealed highest PPO activities (46.0–55.2 nkat/mL) at 30–40 °C in a range of pH 6.0–6.5 after addition of 0.5 g/g_sample_ polyvinylpyrrolidone and 0.5% (*v*/*v*) Triton X-100 during extraction. ‘Cavendish’ PPO activity was four times higher. Banana peel PPO exhibited the highest affinity towards dopamine (*K*_M_ = 0.94 mM). Thermal inactivation of ‘Prata’ and ‘Cavendish’ PPO was achieved at 90 °C after 5 and 15 min, respectively, whereas cold plasma treatment did not decrease PPO activity below 46% of the initial enzyme activity. The inactivation behavior of PPO could successfully be described by a two-fraction model indicating at least two types of isoenzymes with different thermal stability. The overall high thermal stability was mainly attributed to membrane-bound PPO. The results may help to prevent enzymatic browning of banana peels and thereby facilitate their valorization as food ingredients.

## 1. Introduction

Bananas are amongst the economically most important fruits worldwide. Even though the Cavendish subgroup dominates international markets, the banana cultivar ‘Prata’ (*Musa acuminata* x *M. balbisiana*) has widespread availability in the Brazilian market with rising sales [1]. Considering the mass proportion of the peels, which accounts for approximately 40% of the fresh fruit weight, it is evident that the processing of bananas into purees, chips, or other dried products results in huge amounts of residues [2]. In view of the increasing importance of sustainability in food production, strategies for the valorization of side streams are urgently required to reduce the amount of waste and hence the environmental burden that would arise from disposal of by-products [3].

Banana peels represent a promising source of dietary fiber [2] and secondary plant metabolites, e.g., phenolic compounds [4]. Despite their promising composition, the implementation of by-product utilization in industrial banana processing is still limited. Besides microbiological spoilage and the potential presence of pesticide residues, quality loss because of enzymatic browning is considered one of the main challenges. One of the enzymes associated with enzymatic browning in fruits and, in particular, in bananas, is polyphenol oxidase (PPO) [5]. PPO is a group of enzymes that can be divided into monophenol monooxygenases (EC 1.14.18.1) and *o*-diphenoloxidases (EC 1.10.3.2). While the first category catalyzes the oxidation of monophenols to *o*-diphenols, the latter oxidize *o*-diphenols to *o*-quinones in the presence of oxygen [6]. The quinones irreversibly and rapidly polymerize or react with proteins and amino acids, resulting in high molecular brown-black pigments. If no countermeasures are taken, losses in nutritive value and brown discoloration of fruits will occur [7].

Besides the application of anti-browning agents, suffering from a declining acceptance of the consumers, thermal treatment is the most dependable approach to inactivate fruit enzymes and, thus, overcome enzymatic browning [8]. However, quality deterioration by degradation of phytochemicals and other constituents can arise from thermal food preservation [4,9]. Therefore, the food industry is searching for alternate technologies that enable enzyme inactivation while retaining their nutrients, flavor, and appearance. Among others, cold atmospheric pressure plasma (CAPP) treatment is considered as a non-thermal alternative for enzyme inactivation [10]. Plasma can be generated by applying energy sufficient to ionize a neutral gas. Non-thermal (non-equilibrium) plasma, also referred to as cold plasma, consists of reactive oxygen species (ROS) and reactive nitrogen species (RNS), ions, electrons, radicals, UV light, and other reactive constituents. CAPP has been known as a technology for the decontamination of surfaces, inactivating both bacteria and spores [11]. In addition, the inactivation of PPO has recently been observed in apple juice and coconut water [12], as well as in fresh-cut apples [13] and potatoes [14]. Especially indirect plasma treatment with plasma processed air (PPA) under atmospheric pressure is of interest, as fresh produce are not exposed to elevated temperatures [14].

To effectively control the enzymatic browning during the post-harvest processing of banana peels, a deeper understanding of the PPO present in banana peels of different varieties and their affinity towards different substrates is essential. Whereas banana pulp PPO of different cultivars have been characterized [15,16,17,18,19,20,21,22,23], only partial characterization of substrate specificities and effects of inhibitors of *M. acuminata* peel PPO [22,23] and a comprehensive characterization of an unspecified *M. sapientum* L. peel PPO [5] have been reported. However, varietal differences are a common phenomenon amongst fruits [24,25,26]. Similarly, differences in PPO activities [19,21] and PPO characteristics, namely substrate and inhibitor specificity [22], were reported for different banana tissues. Stressing the importance of understanding the PPO characteristics of different relevant banana varieties and stating the fact that ‘Prata’ banana peel PPO has not yet been characterized, a gap in previous research can therefore be identified. Thus, the pH and temperature optima, the substrate specificity, and the thermal stability of PPO from ‘Prata’ peels were studied and the results will be presented in this work.

Two major aspects have to be considered during the extraction of PPO from phenol-rich sources like banana peels [25]. First, the oxidation of phenolic compounds, which are found in high quantities in banana peels [4], needs to be limited to prevent browning reactions. Second, as PPO can be present in membrane-bound and free form [6,25,27,28], complete solubilization of the PPO needs to be assured to make a general statement about the PPO characteristics and inactivation behavior. While increasing the ionic strength is a gentle alternative to solubilize weakly membrane-bound PPO, the addition of detergents may be required to extract tightly bound PPO [27].

In the present study, the main objective was to evaluate the potential of different processes on the inactivation of PPO in a crude enzyme extract of banana peels obtained from two varieties (‘Cavendish’ and ‘Prata’) to enable subsequent prevention of enzymatic browning. For this purpose, an adapted extraction protocol for banana peel PPO was established along with characterizing substrate specificity and optimum reaction conditions. Furthermore, the stability of PPO towards thermal and PPA treatment was examined, and inactivation kinetic parameters were derived. These parameters are required to predict suitable process conditions and thereby enable the efficient design of PPO inactivation treatment. This work provides (i) information about the extraction and characterization of ‘Prata’ banana peel PPO, (ii) a comparison of thermal stability of different dessert banana PPO, (iii) insights into the inactivation kinetics of thermally treated crude, soluble, and membrane-bound banana peel PPO, and (iv) data about the inactivation kinetics of banana peel PPO treated with CAPP, for the first time.

## 2. Materials and Methods

### 2.1. Material and Reagents

Brazilian ‘Prata’ bananas (*Musa acuminata* x *M.balbisiana*, genomic group AAB, Latitude 9 Exportação, Petrolina PE, Brazil) and ‘Cavendish’ bananas (*M. acuminata*, genomic group AAA, Union de Bananeros Organicos Inmaculada Concepcion, Huangalá–Sullana, Piura, Peru) were used as raw material. At least 1 kg of bananas was washed. The fruit apex and the stem were removed before the fruits were peeled. The peels of each cultivar were pooled and pre-comminuted with a knife. After freezing the peels at −50 °C they were placed in a freeze dryer (LMC-2, Christ Gefriertrocknungsanlagen GmbH, Osterode am Harz, Germany) for 48 h. The pressure was adjusted to 1.030 mbar, and the condenser temperature was set to −55 °C. The freeze-dried peels were ground in a Grindomix GM 200 knife mill (Retsch GmbH, Haan, Germany) for 20 s to obtain banana peel flour and stored at −20 °C until PPO extraction.

All chemicals used were of analytical grade and were purchased from Sigma-Aldrich (Merck KGaA, Darmstadt, Germany), except for disodium hydrogen phosphate and citric acid, which were sourced from Merck (Merck KGaA, Darmstadt, Germany), bovine serum albumin (BSA) and Bradford dye, which were from Bio-Rad (Bio-Rad Laboratories GmbH, Feldkirchen, Germany), and polyvinylpolypyrrolidone (PVPP) obtained from Honeywell Fluka (Fisher Scientific GmbH, Schwerte, Germany).

### 2.2. Preparation of Crude Enzyme Extracts

Previously described extraction protocols applied for banana pulp and apple PPO were specifically adapted to meet the requirements for determining PPO activity in banana peels [19,25]. For this purpose, 1 g of the powdered sample was mixed with chilled McIlvaine buffer (pH 6.5; 4 °C) consisting of 0.1 M citric acid and 0.2 M disodium phosphate, at a liquid-to-solid ratio of 30:1, unless stated otherwise. To adapt the PPO extraction method, different filter aids (PVP and PVPP) and concentrations of Triton X-100 were used. Besides, liquid-to-solid ratios from 30:1 to 50:1, extraction times from 0 to 24 h, and homogenization times from 0 to 6 min were varied, as displayed in Table 1. Varying quantities of polyvinylpyrrolidone (PVP) or PVPP were blended with the dry sample before buffer addition. The resulting suspension was homogenized with an Ultra-Turrax T25 homogenizer (IKA-Werke GmbH & Co. KG, Staufen, Germany) at 3500 rpm on ice. Subsequently, PPO extraction was carried out for up to 24 h at 4 °C under constant stirring of 300 rpm on a magnetic stirrer (IKA Werke GmbH & Co. KG). The resulting suspension was centrifuged at 20,000× *g* for 20 min at 4 °C (Sigma 3K30, Sigma Laborzentrifugen GmbH, Osterode am Harz, Germany). Supernatants were collected after filtration through Whatman No. 1 filter paper (GE GmbH, Freiburg, Germany) and stored as aliquots at −20 °C for up to two weeks. Examined extraction parameters were changed according to a one-factor-at-a-time model. All extractions were performed in triplicate.

### 2.3. Fractionation of Crude Enzyme Extracts

To extract fractions of soluble (sPPO) and membrane-bound PPO (mPPO), a method described by Zhou et al. [28] and Zaini et al. [6] for the fractionation of pear and snake fruit PPO was applied with slight modifications. Briefly, sPPO was extracted from 1 g of dried banana peel flour with 0.5 g of PVP (optimal dosage determined in our experiments) in 30 mL of McIlvaine buffer (pH 6.5) as described before. While the supernatant was used as crude sPPO extract, the pellet was subjected to an additional extraction step to obtain a crude mPPO extract. For this purpose, the pellet was suspended in 30 mL of 0.1 M Tris buffer, pH 6.8, containing 1.5% (*v*/*v*) Triton X-114. After sonication in a Sonorex Digitec DT 100 H ultrasonic bath (Bandelin electronic GmbH & Co. KG, Berlin, Germany) for 10 min, the sample was centrifuged again as described above. The resulting supernatant was then subjected to a temperature-induced phase partitioning. While increasing the concentration of Triton X-114 to 8% (*v*/*v*), the temperature was kept constant at 4 °C for 30 min. After heating the sample to 35 °C for 15 min, the fluid turned turbid due to the formation of mixed micelles containing hydrophobic compounds. Centrifugation at 20,000× *g* for 20 min at 25 °C resulted in a detergent-rich phase and a clear supernatant, with the latter containing peripheral membrane proteins being used as crude mPPO extract.

### 2.4. Protein Determination of Crude Enzyme Extracts

The protein content of the crude enzyme extracts was determined using an assay kit (Bio-Rad Protein Assay) adapted from Bradford [29] with bovine serum albumin (BSA) as an external standard. Bovine serum albumin (BSA) concentrations for the calibration curve were in the range of 10 to 75 µg/mL. Aliquots of 50 µL of standard solution or enzyme extract were pipetted into a well of a polystyrene microplate. Subsequently, 200 µL of a 20% (*v*/*v*) dye concentrate diluted in demineralized water and filtered through Whatman No. 1 filter paper was added. After incubating for 10 min at room temperature, the absorbance was measured at 595 nm with a microplate spectrophotometer (µQuant MQX 200, BioTek Instruments, Winooski, VT, USA). All solutions were analyzed in triplicate.

### 2.5. PPO Activity Assay

The PPO activity was determined spectrophotometrically according to Schweiggert et al. [30]. Unless stated otherwise, the reaction mixture consisted of 1125 µL McIlvaine buffer (pH 6.5), 150 µL of 0.5 M L-proline in McIlvaine buffer (pH 6.5), and 75 µL of crude enzyme extract. Subsequently, 150 µL of 25 mM 4-methylcatechol in McIlvaine buffer (pH 6.5) was added to start the formation of a pink proline-catechol reaction product. The resulting increase in absorption was monitored every 15 s for 4 min at 525 nm against a reagent blank without enzyme extract with a Specord 210 PLUS spectrophotometer (Analytik Jena AG, Jena, Germany). A PTC 800 Peltier element (Analytik Jena AG) allowed temperature-controlled kinetic measurements at a given temperature. The linear increase in absorption during the first 60 s and a molar extinction coefficient of ε = 1550 L mol^−1^ cm^−1^ were used to quantify the PPO activity. All measurements were carried out in duplicate.

### 2.6. Characterization of Crude PPO

To further characterize ‘Prata’ banana peel PPO, the pH optimum of the crude PPO was determined in McIlvaine buffers ranging from pH 4 to 8. The pH was adjusted by adding 0.1 M citric acid to 0.2 M Na_2_HPO_4_ until the respective pH was reached. The enzyme activity was measured at 25 °C.

The optimal temperature of the crude PPO was determined in McIlvaine buffer at optimal pH (pH 6.5) in the temperature range from 20 to 70 °C. Reaction buffer and L-proline were incubated in an Eppendorf Thermomixer comfort (Eppendorf AG, Hamburg, Germany) at 600 rpm at respective temperatures. After adding the enzyme extract, 4-methylcatechol was added, and enzyme activity was measured.

The substrate specificity of PPO was tested for selected mono- and diphenols. Dopamine and chlorogenic acid were diluted in McIlvaine buffer (pH 6.5) and added to the reaction mixture to obtain a final concentration in the range of 0.25 to 10 mM. Due to the limited solubility of L-tyrosine, final concentrations in McIlvaine buffer were between 0.25 and 2 mM. PPO activity was determined at the absorption maximum of each reaction product, which was 475, 400, and 400 nm for dopamine, chlorogenic acid, and L-tyrosine, respectively.

In addition, the effect of the concentration on the coupling reaction of 4-methylcatechol and L-proline, which leads to the formation of 5-methyl-4-*N*-prolyl-*o*-benzoquinone, was studied. For this purpose, the concentration of 4-methylcatechol in the final reaction mixture was varied in the range of 0.25 to 10 mM, while a constant L-proline concentration was maintained.

PPO activity and respective substrate concentrations were used to estimate the Michaelis–Menten constant (*K*_M_) and the maximum velocity of reaction (*V*_max_) according to the Michaelis–Menten equation by non-linear regression with OriginPro 2018b (OriginLab Corp., Northhampton, MA, USA). The Michaelis–Menten constant *K*_M_ and the maximum velocity *V*_max_ represent the PPO’s affinity against a substrate and the maximum product formation rate. The substrate conversion efficiency, or catalytic power (*V*_max_/*K*_M_), was calculated from the resulting values.

### 2.7. Thermal PPO Inactivation in Crude Enzyme Extracts

Crude PPO extracts in Eppendorf tubes were incubated in a Thermomixer comfort at 300 rpm. Temperatures between 60 and 90 °C were chosen for incubation times between 1 min and 24 h to investigate the thermal PPO inactivation kinetics. The tubes were transferred into an ice bath immediately after the heat treatment, and residual PPO activity was measured according to Section 2.5. All experiments were conducted at least in triplicate.

### 2.8. Plasma Treatment of Crude Enzyme Extracts

Plasma treatments of the crude PPO extracts were conducted with a portable plasma system (Plasmatreat GmbH, Steinhagen, Germany) described by Kramer et al. [11] with slight modifications. Plasma was produced using a plasma nozzle based on a dielectric barrier discharge plasma source with a discharge frequency of 13,500 Hz and a power input of 200 W. A carrier gas stream (7.5 L/min) comprising of ambient air at 6 bar was directed through the plasma nozzle into an adjacent glove box (0.5 m × 0.6 m × 1 m), where the treatment took place. Ozone-resistant gloves enabled the handling of the samples inside the box. The plasma-processed air (PPA) was humidified via carrier gas stream by introducing water vapor into the plasma afterglow. The water vapor was produced by pumping water over an evaporator at 150 °C at a flow rate of 1 mL/min. Two Petri dishes with a diameter of 90 mm, each containing 5 mL of the crude PPO extracts, were placed on a sample table inside the glove box and treated for 1 min to 30 min. Prolonged treatment was achieved by subsequent treatment intervals of 30 min to avoid overheating of the plasma nozzle. The PPO extracts were cooled immediately after the treatment and analyzed for their residual activity on the same day.

### 2.9. Kinetic Data Analysis

After specified treatment times, residual activities of the PPO were used for the estimation of parameters via non-linear regression using non-linear least-squares analysis by OriginPro 2018b. In order to describe the inactivation behavior of banana peel PPO, different models were used.

First-order kinetics (Equation (1)) is the most common model applied for the description of the influence of thermal treatment on enzyme activity [7,15,31] and is related to the enzyme activity after a specific treatment time relative to the initial enzyme activity as follows:

(1) $$\frac{A_{t}}{A_{0}}=\exp\left(-kt\right)$$

where *A_t_* and *A*_0_ are defined as enzyme activity of the treated crude PPO after time *t* and the native PPO, respectively, *t* is the treatment time, and *k* is the inactivation rate constant.

Considering that several isoenzymes with different stabilities might be present in the crude PPO extract, biphasic models such as the two-fraction model can also be used to describe the inactivation behavior of enzymes. The two-fraction model displayed in Equation (2) was used to model inactivation kinetics of enzymes such as PPO [24,32].

(2) $$\frac{A_{t}}{A_{0}}=\alpha \exp\left(-k_{L}t\right)+\left(1-\alpha \right)\;\exp\left(-k_{S}t\right)$$

where *A_t_* and *A*_0_ are the enzyme activities of the treated and the native crude PPO, *t* is the treatment time, *k_S_* and *k_L_* are inactivation rate constants of the stabile and labile fraction, and *α* represents the active fraction of the labile isoform in relation to the total activity.

Additionally, the Weibull distribution model, according to Equation (3), was evaluated regarding the approximation of the inactivation kinetic.

(3) $$\frac{A_{t}}{A_{0}}=\exp\left(-bt^{n}\right)$$

where *A_t_* and *A*_0_ are the enzyme activities of the treated and the native crude PPO, *t* is the treatment time, *b* is the scale parameter, and *n* is the shape parameter. In contrast to Equations (1) and (2), the Weibull model is based on an empiric rather than a kinetic approach. Empiric models tend to be more flexible as they do not follow physical laws [12,33].

The fit of the models was evaluated in terms of the suitability of the model to describe the variance of the measured values and the difference of measured values from the predicted values using the adjusted coefficient of determination (R^2^_adj_) and root-mean-square error (RMSE), respectively [34]. Besides, an uncorrelated, random distribution of residuals was considered an indicator for a suitable model for the inactivation of banana peel PPO [35].

### 2.10. Statistical Analyses

All values are expressed as the mean of at least three experiments (*n* = 3) ± standard deviation. Statistical analysis was performed by one-way analysis of variance (one-way ANOVA) if the normality test (Shapiro–Wilk test) was passed. Means were compared using the Tukey test (*p* < 0.05) to determine significant differences amongst samples.

## 3. Results

### 3.1. Adaption of PPO Extraction and Characterization of Temperature and pH Optimum of ‘Prata’ Banana Peel PPO

#### 3.1.1. Adaption of PPO Extraction

Crude PPO extract obtained from Brazilian ‘Prata’ banana peels extracted with McIlvaine buffer lacking any additives showed a low initial PPO activity of 36.6 nkat/mg_protein_, indicating unspecific and incomplete PPO extraction (Table 2). Therefore, different parameters of the extraction protocol were modified to increase the extraction of PPO and, thus, include a higher percentage of the banana peel PPO isoenzymes in the subsequent characterization and inactivation studies. While the extraction adaption was conducted for ‘Prata’ banana peel PPO, a crude PPO extract was also prepared from ‘Cavendish’ peels under the optimum extractions conditions determined in this study to demonstrate the applicability of the adapted PPO extraction regime for peels of the other banana variety.

Specific PPO activity in crude enzyme extracts could solely be increased significantly (*p* < 0.001) by adding at least 0.5 g PVP to the extraction buffer. The high content of phenolics, which was previously reported for banana peels [4], is a challenge during the extraction of PPO from plant sources because oxidized and polymerized phenolic compounds can lead to conformational change and inactivation of the PPO [36]. Beneficial effects of adding phenol binding PVP or PVPP on the extractability of PPO from eggplant [27], mango [26], and banana root [37] have been reported previously. Moreover, the volumetric PPO activity could be increased significantly as a response to the addition of 0.5 g Triton X-100 to the extraction buffer, which indicates the presence of a strongly membrane-bound PPO form [27].

At the same time, no significant differences of specific PPO activities were observed (*p* ≥ 0.398) after the addition of Triton X-100, which indicates unspecific overall solubilization of membrane-bound proteins resulting in increased protein contents of the crude PPO extracts. Varying liquid-to-solid ratio (*p* ≥ 0.237), extraction time (*p* ≥ 0.37), and homogenization time (*p* ≥ 0.22) had no significant influence on the specific and volumetric PPO activities. However, prolonged extraction time of 24 h at 4 °C resulted in a significant specific PPO activity loss (*p* = 0.035) compared to extraction times between 0 and 10 h in the presence of 0.5 g PVP and 0.5% (*v*/*v*) Triton X-100. Additionally, homogenization using an UltraTurrax did not significantly increase PPO activity, which is in contrast to previous findings displaying the necessity for homogenization when using fresh banana samples [5,16,19].

Differences in volumetric and specific PPO activities amongst the controls of the individual series (banana peels extracted without any filter aids and detergent) might be explained by variations of the raw materials, despite the use of banana peels with the maturity degree 7 (yellow with a few dark spots) determined by the color chart [2] and a pooling of the dried peels before the extraction experiments. In particular, this was observed for the specific PPO activity to a greater extent as the protein content of the crude enzyme extracts varied, thus increasing this variation. Therefore, complete solubilization of the PPO by using detergents such as Triton X-100 is even more important to ensure consistent PPO activity in the banana peel extract.

In conclusion, the extraction of 1 g lyophilized banana peels in 30 mL McIlvain buffer (pH 6.5) with the addition of 0.5 g PVP and 0.5% (*v*/*v*) Triton X-100 was carried out without any homogenization or extraction step. Thus, specific PPO activities of 143.6 and 269.1 nkat/mg_protein_ were achieved for crude PPO extracts from ‘Prata’ and ‘Cavendish’ peels at the adapted extraction procedure, equaling to 222.5 and 417.1 Units/mg_protein_, respectively, with one unit of enzyme activity being defined as a change in absorbance of 0.001 per min. In comparison to the specific activity of *M. sapientum* peel PPO of 2250 Units/mg_protein_ reported by Yang et al. [5], our activities were lower, which may be attributed to both the use of different varieties and different PPO activity assay procedures.

#### 3.1.2. Temperature and pH Optimum of Crude ‘Prata’ Banana Peel PPO

**Figure 1:**
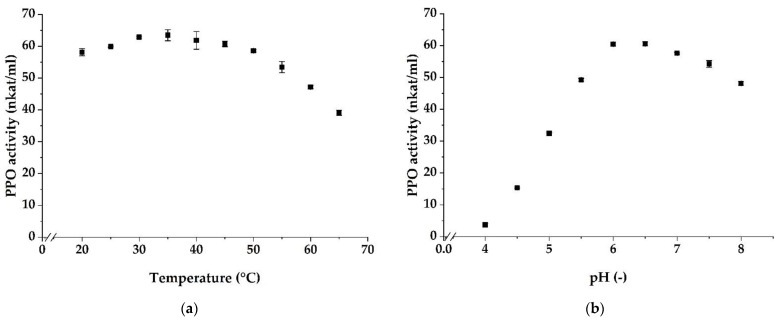
Effect of temperature (**a**) and pH (**b**) on ‘Prata’ banana peel polyphenol oxidase (PPO) activity (nkat/mL). Values are expressed as mean ± SD of three experiments.

The temperature dependence of ‘Prata’ banana peel PPO activity was determined at different temperatures in the range of 20 to 65 °C using 4-methylcatechol as the substrate and L-proline as the coupling agent at pH 6.5. ‘Prata’ banana peel PPO revealed an optimum temperature within the range of 30 to 40 °C (Figure 1a), which is in agreement with findings for PPO extracted from dessert banana *M. acuminata* cv. ‘Anamur’ pulp [15], and *M. sapientum* L. peels [5] and pulp [17]. In addition, the observed temperature optimum of ‘Prata’ banana peel PPO is within the same range as for other plant PPO [6,7].

The pH-dependent PPO activity profile for the oxidation of 4-methylcatechol was measured at different pH values from 4 to 8 (Figure 1b). The PPO showed maximum activity at pH 6 to 6.5, which is in good agreement with previously reported pH optima in the range of 6 to 8 of dessert banana peel PPO [5] or banana pulp PPO [15,16,17]. Therefore, only minor deviations seem to occur for banana peel and pulp PPO regarding their temperature and pH optima. Nevertheless, the temperature and pH optimum of PPO can drastically be affected by fruit cultivar, plant part, and growing conditions, as shown for mango PPO [26].

#### 3.1.3. Substrate Specificity and Kinetic Parameters—Oxidation of Phenolic Compounds

Substrate specificity of PPO depends on the plant source and the cultivars [24]. Therefore, the conversion of the three phenolic compounds dopamine, chlorogenic acid, and L-tyrosine was tested to evaluate the substrate specificity of ‘Prata’ banana peel PPO since previous studies on enzyme kinetics of banana peel PPO have been limited to dopamine [5]. Additionally, the coupling reaction of L-proline to the oxidized phenolic compound 4-methylcatechol was evaluated for its potential to increase the sensitivity of the PPO activity assay. The assay was conducted within the identified pH and temperature optimum (pH 6.5, 30 °C).

‘Prata’ banana peel PPO showed the highest activity towards dopamine (Table 3), whereas the activity towards chlorogenic acid was comparatively low, even though it is a widespread plant metabolite. No volumetric PPO activity towards the monophenolic compound L-tyrosine could be detected. An absence of monophenol monooxygenase activity has been observed previously for banana pulp [18,20] and peel PPO [22].

**Figure 2:**
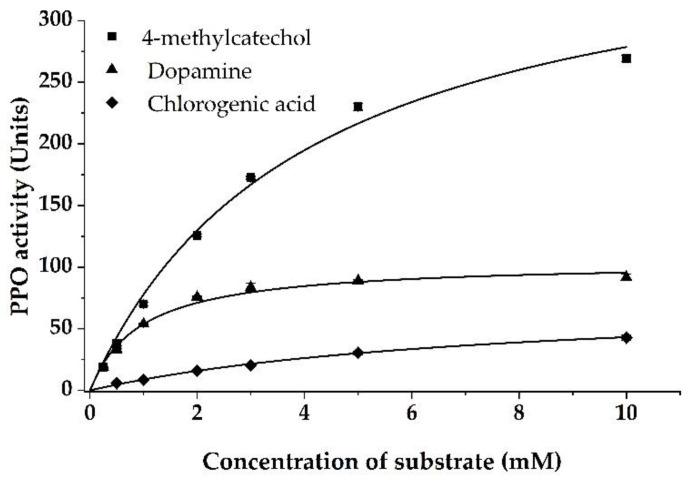
Michaelis–Menten kinetics of the ‘Prata’ banana peel polyphenol oxidase (PPO) in McIlvaine buffer (pH 6.5) for dopamine, chlorogenic acid, and 4-methylcatechol reacting with 50 mM L-proline. Values are expressed as mean ± SD of three experiments. One unit is defined as a change of absorption of 0.001 ∆A per minute.

For the tested diphenolic substrates dopamine and chlorogenic acid the ‘Prata’ banana peel PPO followed Michaelis–Menten kinetics (Figure 2). The lowest *K*_M_ and hence the highest affinity was observed for dopamine (0.94 mM), while a *K*_M_ of 7.54 mM was shown for chlorogenic acid. Moreover, the maximum product formation rate represented by a *V*_max_ of 104.5 Units was higher for dopamine. Thus, the resulting substrate conversion efficiency of crude banana peel PPO for dopamine was 110.8 Units/mM, which is more than ten times higher than the one observed for chlorogenic acid with 10.4 Units/mM.

The observed *K*_M_ value for dopamine is in a similar range as the values reported for PPO from banana peels [5], banana pulp [16,17,18], and banana root [37]. The *K*_M_ value of ‘Prata’ banana peel PPO for chlorogenic acid was twice as high as the *K*_M_ of a dwarf banana pulp PPO (4.1 mM) [18]. However, the observed value was higher than 0.34–0.37 mM obtained for avocado PPO [24], reflecting a lower affinity of ‘Prata’ banana peel PPO to chlorogenic acid as a substrate. The maximum product formation rate for dopamine and chlorogenic acid were slightly higher than reported *V*_max_ of 0.085 and 0.062 ∆A per min, corresponding to a *V*_max_ of 85 and 62 Units for banana root PPO [37]. The lower catalytic power towards chlorogenic acid, which is in agreement with a lower relative activity observed previously for ‘Cavendish’ banana pulp and peel PPO [22], might be explained by the molecular structure of chlorogenic acid, which consists of an ester of caffeic and quinic acid and, therefore, has a larger substituent at the phenolic ring compared to dopamine. Besides, due to its presence in a relatively high amount of up to 560 and 10 mg/100 g in banana peels and pulp [4], the high affinity of ‘Prata’ banana peel PPO towards dopamine supports the hypothesis that dopamine is an endogenous substrate in various banana tissues and cultivars [5,38].

#### 3.1.4. Substrate Specificity and Kinetic Parameters—Adduct Formation of 4-Methylcatechol and L-Proline

To increase the sensitivity of the PPO assay, the product of the coupling reaction of the oxidized diphenol 4-methylcatechol and L-proline was also used to quantify the PPO activity [30]. Similar to the phenolic substrates tested in this study, it could be shown that the enzymatic reaction of ‘Prata’ banana peel PPO followed Michaelis–Menten kinetics (Figure 2) when keeping the concentration of L-proline constant at varying 4-methylcatechol concentrations. Based on the concentration-dependent PPO activity, an apparent *K*_M_, *V*_max_, and substrate conversion efficiency of 4.01 mM, 390 Units, and 97.3 Units/mM were observed, respectively. Comparing the apparent *K*_M_ value for the oxidation of 4-methylcatechol in the presence of L-proline to previous investigations using solely 4-methylcatechol, our result is half of the *K*_M_ value obtained for banana root PPO (8.3 mM). In contrast, the obtained apparent *V*_max_ is comparable to the reported *V*_max_ of 0.417 ∆A per min, corresponding to 417 Units, for banana root PPO [37]. Because the oxidation of two molecules of 4-methylcatechol results in the formation of one molecule of 5-methyl-4-*N*-prolyl-*o*-benzoquinone [39], the apparent *K*_M_ value of the adduct formation is comparable to the values obtained without L-proline as a coupling agent. Additionally, the apparent *V*_max_ of the proline-coupled assay is reduced by one-half compared to the direct assay. Thus, our results for the proline-coupled assay indicated a higher affinity and a higher maximum product formation rate of banana peel PPO in comparison to banana root PPO [37].

However, due to the high apparent maximum product formation rate, the catalytic power of the banana peel PPO did not differ significantly (*p* = 0.308) for dopamine and 4-methylcatechol. The sensitivity, indicated by the volumetric PPO activity, was increased to 1673 Units/mL by the coupling reaction of 4-methylcatechol and L-proline probably due to the reaction product’s high extinction coefficient of ε = 1550 L mol^−1^ cm^−1^ [30,39].

### 3.2. Temperature Stability of Crude Banana Peel PPO

**Figure 3:**
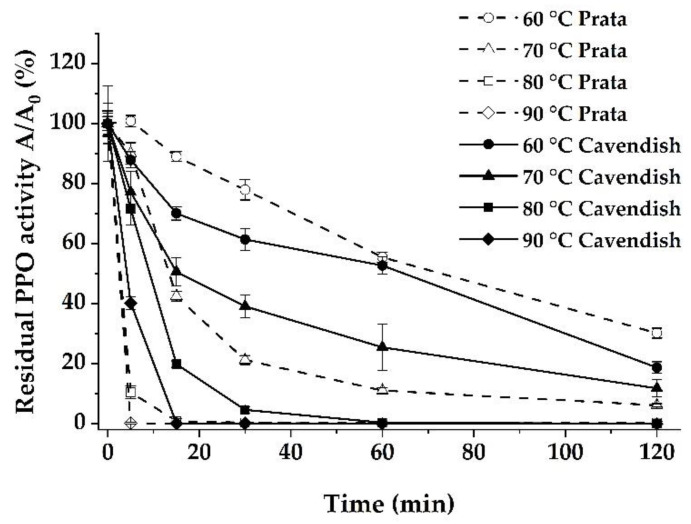
Effect of thermal treatment on residual PPO activity (%) of crude ‘Prata’ banana and ‘Cavendish’ peel polyphenol oxidase (PPO). Values are expressed as mean ± SD of three experiments.

The temperature stability profiles of the PPO extracted from ‘Prata’ and ‘Cavendish’ banana peels were investigated for temperatures between 60 and 90 °C (Figure 3). Comparing the initial PPO activities of the crude PPO extracts obtained under ideal extraction conditions for the two different banana cultivars, the volumetric PPO activity of ‘Cavendish’ peels is approximately four times higher than that of the ‘Prata’ cultivar (46.0–55.2 nkat/mL) with PPO activities in the range of 204.8–211.9 nkat/mL. Due to the significantly different initial PPO activity levels of the crude PPO from the two banana varieties, the values were normalized, and the residual activities after thermal treatments were calculated.

In general, increasing the temperatures and times resulted in a decreased residual PPO activity of crude PPO extracts from both peels, exhibiting similar inactivation curves. At 60 °C, an almost linear decrease in residual PPO activity was observed, whereas at higher temperatures, the inactivation was fast at short treatment times, while the inactivation velocity decreased over time. For instance, at 60 and 70 °C residual PPO activities were above 5% even after treatment for 120 min. Accordingly, the inactivation of crude peel PPO was insufficient for both banana varieties. In contrast, an almost complete PPO inactivation below 0.5% residual activity was achieved for the thermally more stable ‘Cavendish’ cultivar after incubation at 80 °C for 60 min or at 90 °C for 15 min, respectively.

A comparison of the thermal stability of the two cultivars revealed that the treatment of the crude PPO extracts above 60 °C resulted in a faster decrease of ‘Prata’ banana peel PPO activity compared to ‘Cavendish’ banana peel PPO. However, ‘Prata’ banana peel PPO showed less susceptibility to thermal treatment at 60 °C. Therefore, it seems that the ‘Prata’ banana peel PPO contains a higher ratio of thermally stable PPO, which, in turn, is more easily inactivated than the thermally stable ‘Cavendish’ peel PPO.

Chaisakdanugull et al. [16] reported inactivation of ‘Gros Michel’ banana pulp PPO after 5 min treatment at 90 °C, which indicates lower thermal stability of pulp PPO compared to peel PPO. However, the differences might also be attributed to different varieties as well as a lower degree of maturation. Besides, the crude PPO extracts probably contain other substances, e.g., other cellular proteins, which might stabilize the active conformation of the PPO and thus lead to a higher apparent thermal stability [24]. Even though these results are consistent with the thermal stability of coconut water PPO [31], they significantly differ from blueberry PPO, which can be inactivated by a 2 min treatment at 80 °C [9]. These findings indicate a high thermal stability of banana peel PPO. Because of the higher thermal stability and thus the greater challenge of inactivating crude PPO, the inactivation kinetics were evaluated for crude ‘Cavendish’ banana peel PPO and related to the presence of soluble and membrane bound PPO.

### 3.3. Kinetics of Crude PPO Thermal Inactivation

Based on the thermal stability data of crude ‘Cavendish’ banana peel PPO, different models were evaluated regarding their potential to describe the inactivation kinetics. Considering the estimated parameters of first-order models obtained by non-linear regression (Table 4), an increasing inactivation rate constant k from 0.014 min^−1^ at 60 °C to 0.197 min^−1^ at 90 °C was observed, reflecting an increasingly fast inactivation.

**Figure 4:**
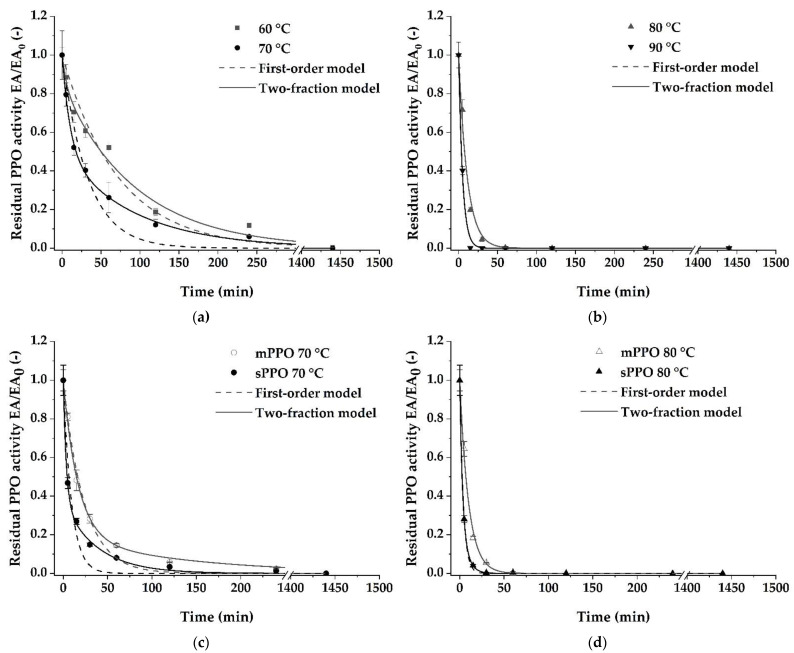
Comparison of first-order (dashed line) and two-fraction model curve fitting (solid line) for the thermal inactivation of ‘Cavendish’ banana peel polyphenol oxidase (PPO): (**a**) crude PPO at 60 and 70 °C, (**b**) crude PPO at 80 and 90 °C, (**c**) soluble (sPPO) and membrane-bound PPO (mPPO) at 70 °C, and (**d**) soluble (sPPO) and membrane-bound PPO (mPPO) at 80 °C. Values are expressed as mean ± SD of three experiments.

A high R^2^_adj_ and a low RMSE indicate that the inactivation of the ‘Cavendish’ banana peel PPO at higher temperatures (80 and 90 °C) followed a first-order inactivation model, which is also reflected by a good fit of the curve to the data (Figure 4b). However, the residual activities of the crude PPO were underestimated at 60 and 70 °C, as can be seen from Figure 4a. Therefore, the first-order model cannot reflect the complexity of the thermal inactivation process in the case of crude ‘Cavendish’ banana peel PPO, even though the first-order model is most commonly used for the description of thermal inactivation of PPO from various plant sources like cherries [7], coconut water [31], and banana pulp [15]. Similar findings were described by Padrón et al. [21], who reported an inactivation behavior for banana PPO that deviated from a simple first-order inactivation kinetic without further investigations.

Besides the first-order model, the two-fraction model has been used to describe the biphasic inactivation behavior of thermally treated PPO from radish [32] and avocado [24]. This inactivation model assumes the presence of two fractions with independent inactivation behaviors represented by a stable fraction with a lower inactivation rate constant *k_s_* and a more labile fraction with a higher inactivation rate constant *k_L_*. As shown in Table 4, R^2^_adj_ and RMSE at lower temperatures showed a better representation of the measured values by the two-fraction model than by the first-order model. These findings corroborate our hypothesis that banana peel PPO consists of at least two fractions with different thermal stabilities resulting in a more complex inactivation behavior as already shown for banana pulp PPO by Padrón et al. [21]. Regarding the estimated parameters, the proportion of the temperature-labile fraction α is temperature-dependent, as an increase in the inactivation temperature resulted in an increase of the labile fraction from 15% at 60 °C to 50% at 70 °C and finally 100% at 80 °C, respectively. By increasing the temperature, a higher proportion of the PPO can be considered labile. This temperature dependency indicates that the present PPO is existent in fractions with different temperature stabilities [40]. Since Montgomery et al. [22] found ten isoforms of PPO in banana peels, the varying thermal stabilities might be attributed to both different isoforms as well as to different isoenzymes, which is yet not described in literature for banana peel PPO.

However, the curve fitting of the PPO inactivation at 80 and 90 °C by first-order and two-fraction model were similar (Figure 4b). By increasing the temperature, the time needed to inactivate the labile fraction can decrease to such an extent that the inactivation of the temperature labile fraction is very fast and thus is overlapped by inactivation of the temperature stable fraction. This is also reflected by the estimated proportion of the labile fraction α, which is 1 for the inactivation of the PPO at 80 and 90 °C treatment temperature (Table 4), indicating a pseudo-first-order inactivation. 

Overall, the two-fraction model is appropriate to describe the inactivation behavior of banana peel PPO over the temperature range from 60 to 90 °C, particularly at temperatures of 60 and 70 °C. The obtained inactivation kinetic parameters will assist producers to efficiently develop thermal inactivation processes aiming at preventing enzymatic browning of banana peels. Nevertheless, the underlying mechanisms of this biphasic inactivation behavior still remained unclear and were further investigated by separating soluble (sPPO) and membrane-bound PPO (mPPO) from ‘Cavendish’ banana peels.

### 3.4. Thermal Inactivation Kinetics of Membrane-Bound and Soluble PPO

Sequential extraction of soluble and membrane-bound PPO was applied to ‘Cavendish’ banana peels to investigate the presence of different isoenzymes with diverse thermal stabilities. A similar approach was previously described for apple [41], pear [28], and snake fruit PPO [6], whereby the respective PPOs were divided into a more labile and a more stable fraction by sequential extraction. For this purpose, the mPPO was extracted by temperature-induced phase partitioning after the initial extraction of the sPPO. After confirming that the exchange of detergent did not influence the total PPO activity, Triton X-114 was used instead of the previously applied Triton X-100 because of its lower cloud point. Thus, the temperature during phase partitioning of mPPO could be reduced to 35 °C. Although a slight decrease in PPO activity (17%) was observed after thermal treatment of the crude PPO at 40 °C for 60 min (data not shown), a limited thermal inactivation during fractionation can be expected. The sPPO was the fraction with the higher activity, contributing to 93.5% of total PPO activity, whereas the mPPO made up only 6.5%.

Following the sequential extraction, the two PPO fractions (sPPO and mPPO) were subjected to thermal treatments at 70 and 80 °C. We observed that the mPPO is thermally more stable than the sPPO at both temperatures (Figure 4c,d). For example, treatment of the crude mPPO and sPPO extracts for 15 min at 70 °C resulted in a residual PPO activity of 48.2 and 27.0% and was further decreased to 18.3 and 4.0% after thermal treatment at 80 °C for 15 min, respectively. Han et al. [41] also observed a higher stability of Granny Smith apple mPPO at elevated temperatures, while Zhou et al. [28] found that pear mPPO was more labile than the sPPO in a temperature range from 40 to 75 °C. As suggested by Han et al. [41], the higher temperature susceptibility of banana peel sPPO might result from different quaternary structures of the different PPO fractions.

**Figure 5:**
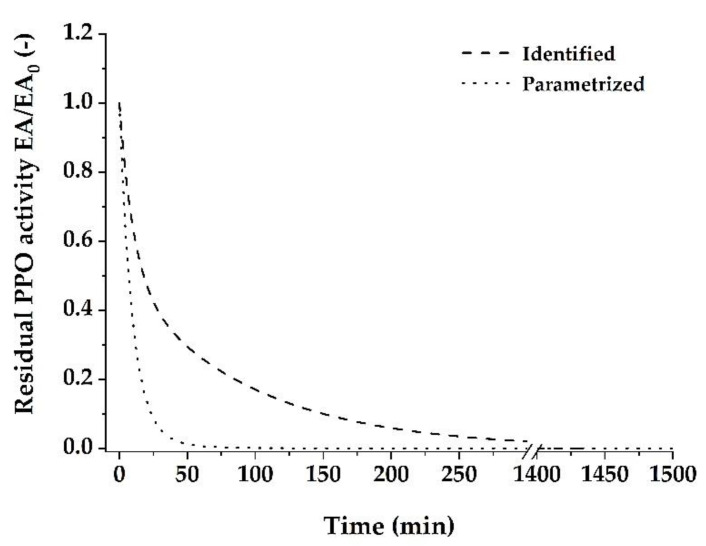
Two-fraction model of the thermal inactivation of ‘Cavendish’ banana peel PPO at 70 °C identified from the data (dashed line) and parametrized based on two individually identified first-order models (mPPO and sPPO) and their respective activity ratio (dotted line).

To determine whether the different thermal stabilities of mPPO and sPPO are responsible for the observed biphasic inactivation behavior of crude banana peel PPO, the parameters of the first-order inactivation kinetics were estimated for both fractions and further used to parametrize a two-fraction model (Figure 5). Similar to the inactivation of crude PPO, the first-order kinetics is suitable to describe the inactivation behavior of sPPO and mPPO, as k shows temperature-dependent behavior (Table 4). However, lower RMSE and patterned residuals at 70 °C of the first-order model reveal that the two-fraction model represents a better approximation of the thermal inactivation behavior of both sPPO and mPPO from ‘Cavendish’ banana peels. Again, the first-order model resulted in an underestimated residual PPO activity over time at 70 °C (Figure 4c). Regarding the estimated parameters of the two-fraction model, overall higher k values were observed for sPPO in comparison to mPPO, indicating higher temperature stability of the mPPO. A temperature increase to 80 °C led to an increased *k_L_* value, while the relative amount of the labile fraction of the mPPO decreased from 81% to 0%. Therefore, the inactivation of mPPO resembles a pseudo-first-order kinetics, which is in accordance with the results of the inactivation trials with the crude PPO extract. Despite this pseudo-first-order inactivation kinetics at 80 °C for crude PPO, two fractions could be observed for the sPPO at both temperatures with a constant relative amount of 63%. However, high uncertainty of the estimated parameters and the similarity of the curve fitting obtained by first-order and two-fraction model (Figure 4d) of sPPO at 80 °C indicates that no biphasic inactivation behavior of sPPO occurred at 80 °C.

Comparing the two-fraction model obtained by parametrizing and the two-fraction model identified from our data illustrates that the sequential extraction could not separate the PPO into two fractions that are responsible for the observed biphasic inactivation behavior of the crude PPO (Figure 5). However, the most surprising observation was that no significant differences between the estimated parameters of mPPO and crude PPO were observed when the inactivation data of crude PPO, sPPO, and mPPO was compared. This similarity indicates that the inactivation of mPPO accounts for the major part of the crude PPO inactivation behavior. In contrast, the inactivation of the sPPO might be secondary, despite its relatively high proportion present in the crude PPO extracts. Nevertheless, considering the thermal inactivation behavior at 70 °C, both the mPPO and the sPPO fraction show biphasic inactivation behavior, which indicates the existence of two isoforms with different thermal stabilities in each fraction. Thus, the crude PPO seems to consist of at least four PPO isoforms. These findings are in contrast to the results of Zaini et al. [6] and Zhou et al. [28]. They reported a first-order inactivation kinetic, indicating each PPO fraction consisted of one isoform. For this reason, we want to stress that our results are motivating to study inactivation kinetics of different fruits and cultivars individually in order to take into account the variability of PPO characteristics.

### 3.5. Effects of Plasma Treatment on PPO Activity

**Figure 6:**
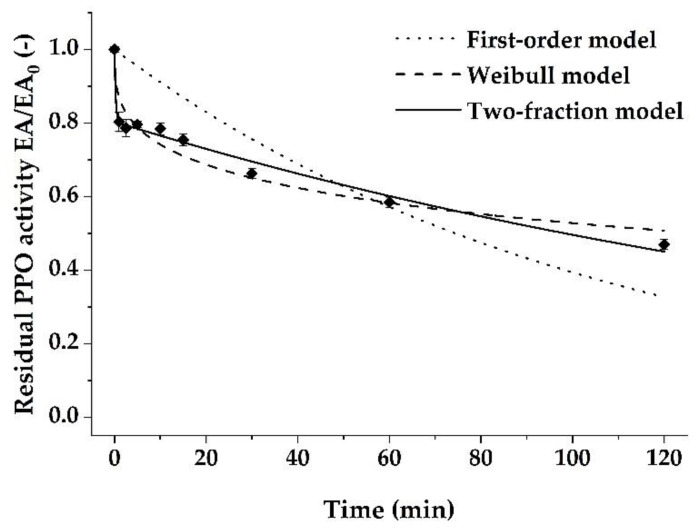
Effect of treatment time on the inactivation of ‘Cavendish’ banana peel PPO by plasma processed air treatment. Values are expressed as mean ± SD of three experiments and continuous lines represent kinetic model data.

Indirect PPA treatment was evaluated for non-thermal inactivation of banana peel PPO. The treatment of crude PPO extracts with PPA resulted in a significant reduction of PPO activity (Figure 6). However, maximal inactivation of 53% of the initial PPO activity was achieved after 120 min treatment at room temperature, whereby a maximum temperature increase of 5 °C was observed. In general, the mode of inactivation by cold plasma treatment depends on the set-up of the inactivation experiment. Therefore, the inactivation might be attributed to reactive species, whereas UV light can be neglected during the indirect treatment applied [10]. Due to a rapid loss of highly reactive species near to the afterglow, an indirect treatment is further characterized by reactions of long-living species like ozone. An involvement of ozone in our inactivation process can be assumed, as an ozone concentration of 2 g/m³ was observed close to the sample surface in a previous trial with a similar experimental set-up [11]. Additionally, the injection of water vapor into the afterglow might have increased the formation of highly reactive water-related species like OH^−^, OH^•^, or H_2_O_2_ [10], but direct interactions with the PPO might be limited due to their fast reactions. However, secondary reactions of stable ROS and H_2_O near the sample, as suggested by Kramer et al. [11], might have led to the formation of hydroxyl radicals. The reactive species formed can oxidize amino acid side chains, cleave peptide bonds, and form peptide-peptide bonds. The resulting conformational changes of the enzyme secondary structure were assumed to cause the loss of activity [42].

The PPO inactivation was characterized by a steep decrease in the residual activity during the first minute of treatment and a lower decrease with prolonged exposure time. A similar inactivation behavior was observed for PPO in PPA treated apple and potato cubes [14]. However, the effectivity of PPO inactivation using PPA differed among studies. While a 10 min exposure of the crude PPO extract to PPA inactivated 22% of the crude banana peel PPO, Bußler et al. [14] reported a 58% and 90% inactivation of apple and potato PPO, respectively, after a 10 min treatment. Direct cold plasma treatment of coconut water PPO [12] was even more efficient as a complete inactivation could be achieved after a 5 min exposure.

As described for coconut water PPO [12], the first-order model with an estimated inactivation rate constant of 0.009 ± 0.001 was not suitable for the description of PPO inactivation caused by CAPP treatment, which is also reflected by an R^2^_adj_ of 0.46 and an RMSE of 0.13 in the present study. The poor fit of this model indicates an oversimplification of the inactivation process, which results in an under-fitted model. Deviations from a simple first-order kinetics might be attributed to different stabilities of PPO isoforms or the complexity of the chemical reactions involved in the inactivation process [10]. For CAPP treatment, the Weibull distribution model has been successfully applied to describe the time-dependent inactivation behavior of PPO [12,33], similar to ozone treatment [34]. The Weibull distribution model is an empirically derived model that reflects the complexity of the enzyme inactivation process by considering several effects simultaneously. With an estimated shape parameter n of 0.33 ± 0.035 and a scale parameter b of 0.003 ± 0.001, an R^2^_adj_ of 0.91 and an RMSE of 0.05 were reached. Even though the Weibull model appears to be a better approach than the first-order model to describe the PPA-induced inactivation behavior of PPO, it is not sufficient to explain the observed inactivation behavior in the present study (Figure 6). Considering the tested models, the biphasic two-fraction model resulted in the best parameter estimates as it explains 94% of the variability of the observed residual PPO activities with a *k_L_*, *k_b_*, and an α of 4.02 ± 8.204 min^−1^, 0.005 ± 0.000 min^−1^, and 0.197 ± 0.014, respectively. RMSE for the two-fraction model was 0.04.

Compared to thermal inactivation, which is based on denaturation caused by elevated temperature, the inactivation effect of PPA treatment results from the reaction between plasma-generated reactive species and PPO. Based on the underlying mechanisms of PPA inactivation, the assumed biphasic inactivation might be ascribed to a limited diffusion depth of the reactive species like OH^•^ into the liquid phase [10]. The initially rapid inactivation expressed as *k_L_* would describe the inactivation of the easily accessible PPO at the surface. At the same time, the PPO below is protected by co-extracted organic constituents of the crude PPO extract like proteins that act as ROS scavengers resulting in a lower *k_s_*. However, the presence of PPO isoforms with differing stabilities cannot be excluded and might be the reason for slight deviations of the measured and predicted residual PPO activities.

## 4. Conclusions

Based on the substrate specificity studies using banana peels, an o-diphenol-oxidase activity (EC 1.10.3.1) was assumed with the highest affinity towards dopamine. As 4-methylcatechol exhibited a similar conversion efficiency like dopamine, and it does not naturally occur in banana peels to a great extent, the coupling reaction of 4-methylcatechol was used to increase the sensitivity of the assay when determining PPO activity in this study. By adapting the extraction protocol for crude banana peel PPO, the volumetric and specific PPO activity could be increased 64.2- and 3.9-fold, compared to the use of McIlvaine buffer without additives. By adapting the extraction process and characterizing the PPO, we could provide optimal extraction and assay conditions for banana peel PPO. A comparison of ‘Cavendish’ and ‘Prata’ cultivars revealed a lower PPO activity and stability of ‘Prata’ banana peel PPO. For this reason, a faster thermal stabilization can be assumed for the latter, which might result in higher retention of nutritional and organoleptic quality.

This study indicates that the application of PPA in the chosen experimental set-up does not appear to be a promising approach for the inactivation of banana peel PPO due to insufficient inactivation, even after long treatment times. In contrast, thermal treatment at high temperatures (80–90 °C) resulted in complete inactivation within one hour or less for ‘Cavendish’ banana peel PPO. As the mPPO seems to predominate the inactivation behavior during thermal treatment, future studies on banana peel PPO should pay attention to solubilize the mPPO during the extraction by utilizing an appropriate detergent. Therefore, the results of the present investigations are the basis for complete utilization of banana peels as high-quality food ingredients and a good source of dietary fibers by inactivating endogenous PPO. Besides, further research is needed to evaluate a potential pesticide accumulation during processing, as well as strategies for the removal of pesticides to ensure food safety and the production of high-quality banana peel ingredients.

## Figures and Tables

**Table 1:** Experiments for the extraction of polyphenol oxidase (PPO) from ‘Prata’ banana peel.

Series	Tested Parameters	Filter Aid (g)		Triton X (% (*v*/*v*))	Liquid/Solid Ratio (mL/g)	Extraction Time (h)	Homogenization Time (min)
		PVP	PVPP				
A	Added filter aid	0, 0.5, 1	1, 2	0	30	4	3
B	Added Triton X-100	0.5	-	0, 0.5, 1	30	4	3
C	Liquid/solid ratio	0.5	-	0.5	30, 40, 50	4	3
D	Extraction time	0.5	-	0.5	30	0, 2, 4, 8, 10, 24	3
E	Homogenization time	0.5	-	0.5	30	0	0, 3, 6

**Table 2:** Influence of extraction conditions on ‘Prata’ banana peel polyphenol oxidase (PPO) activity.

Filter Aid	Triton X-100	Liquid/Solid Ratio	Extraction Time	Homogenization Time	Volumetric PPO Activity	Specific PPO Activity
(g)	(% (*v*/*v*))	(mL/g)	(h)	(min)	(nkat/mL)	(nkat/mg_protein_)
**Series A**						
-	-	30	4	3	0.7 ± 0.1 ^a^	36.6 ± 7.7 ^a^
0.5 g PVP	-	30	4	3	33.5 ± 4.0 ^b^	176.5 ± 19.1 ^b^
1 g PVP	-	30	4	3	35.2 ± 5.1 ^b^	167.1 ± 15.8 ^b^
1 g PVPP ^d^	-	30	4	3	0.2 ± 0.2 ^a^	12.8 ± 14.7 ^a^
2 g PVPP	-	30	4	3	0.2 ± 0.2 ^a^	17.1 ± 9.2 ^a^
**Series B**						
-	-	30	4	3	0.8 ± 0.1 ^a^	46.5 ± 7.5 ^a^
0.5 g PVP	-	30	4	3	26.5 ± 0.6 ^b^	143.9 ± 10.5 ^b^
0.5 g PVP	0.5	30	4	3	58.4 ± 2.2 ^c^	156.0 ± 9.0 ^b^
0.5 g PVP	1	30	4	3	64.7 ± 7.3 ^c^	149.5 ± 8.1 ^b^
**Series C**						
-	-	30	4	3	1.1 ± 0.3 ^a^	66.8 ± 10.3 ^a^
0.5 g PVP	0.5	30	4	3	54.8 ± 9.5 ^c^	143.9 ± 18.3 ^b^
0.5 g PVP	0.5	40	4	3	42.8 ± 1.7 ^b,c^	128.4 ± 6.8 ^b^
0.5 g PVP	0.5	50	4	3	31.0 ± 3.5 ^b^	123.1 ± 10.5 ^b^
**Series D**						
-	-	30	4	3	0.6 ± 0.1 ^a^	31.9 ± 7.5 ^a^
0.5 g PVP	0.5	30	0	3	57.1 ± 3.5 ^b^	165.7 ± 8.6 ^b^
0.5 g PVP	0.5	30	2	3	57.6 ± 7.6 ^b^	168.5 ± 19.2 ^b^
0.5 g PVP	0.5	30	4	3	58.4 ± 2.2 ^b^	156.0 ± 9.0 ^b^
0.5 g PVP	0.5	30	8	3	53.9 ± 0.9 ^b^	151.8 ± 1.3 ^b^
0.5 g PVP	0.5	30	10	3	54.0 ± 0.7 ^b^	154.6 ± 5.1 ^b^
0.5 g PVP	0.5	30	24	3	52.1 ± 3.3 ^b^	122.7 ± 6.7 ^c^
**Series E**						
-	-	30	4	3	0.9 ± 0.2 ^a,*^	56.2 ± 8.3 ^a,*^
0.5 g PVP	0.5	30	0	0	47.5 ± 7.2 ^b^	143.6 ± 12.2 ^b^
0.5 g PVP	0.5	30	0	3	50.1 ± 3.4 ^b^	135.4 ± 11.1 ^b^
0.5 g PVP	0.5	30	0	6	42.7 ± 1.4 ^b^	119.3 ± 8.5 ^b^

Values are expressed as mean ± SD of three experiments. Means in the same column and same series with no letter in common differ significantly (*p* < 0.05). * Mean ± SD of two experiments.

**Table 3:** Polyphenol oxidase (PPO) activity at 2 mM substrate concentration, substrate specificity, and kinetic parameters of ‘Prata’ banana peel PPO for different phenolic substrates. *K*_M_ is the Michaelis–Menten constant, *V*_max_ is the maximum velocity, and *V*_max_/*K*_M_ is the catalytic power.

Substrate	Wavelength	Volumetric PPO Activity	*K*_M_	*V*_max_	*V*_max_/*K*_M_
	(nm)	(Units/mL) *	(mM)	(Units)	(Units/mM)
Dopamine	475	1010.7 ± 6.4	0.94 ± 0.07 ^a^	104.5 ± 2.3 ^b^	110.8 ± 8.9 ^b^
Chlorogenic acid	400	55.8 ± 3.8	7.54 ± 0.51 ^c^	78.7 ± 2.9 ^a^	10.4 ± 0.8 ^a^
L-tyrosine	400	0 ± 0	-	-	-

Values are expressed as mean ± SD of three experiments. Means in the same column with different superscripts differ significantly (*p* < 0.05). * One Unit is defined as a change of absorption of 0.001 ∆A per min at the given wavelength.

**Table 4:** Parameter estimates for the inactivation of crude ‘Cavendish’ banana peel polyphenol oxidase (PPO), membrane-bound PPO (mPPO), and soluble PPO (sPPO) at different treatment conditions. k is the inactivation rate constant, *k_L_* and *k_s_* are the inactivation rate constants of the labile and stabile fraction, respectively, and α is the proportion of the labile isoenzyme fraction.

Model	Parameter	Estimated Values							
		PPO				mPPO		sPPO	
		60 °C	70 °C	80 °C	90 °C	70 °C	80 °C	70 °C	80 °C
**First-order**	k (min^−1^)	0.014 ± 0.001	0.031 ± 0.002	0.091 ± 0.004	0.197 ± 0.007	0.042 ± 0.002	0.100 ± 0.003	0.107 ± 0.010	0.251 ± 0.007
	R^2^_adj_	0.98	0.95	0.99	1.00	0.99	1.00	0.95	1.00
	RMSE	0.05	0.08	0.04	0.02	0.05	0.02	0.07	0.02
	Residual plot	Patterned				Patterned	Patterned		
**Two-fraction**	*k_L_* (min^−1^)	0.177 ± 0.098	0.090 ± 0.023	0.090 ± 0.005	0.195 ± 0.008	0.060 ± 0.006	0.100 ± 0.004	0.290 ± 0.032	0.348 ± 0.522
	*k_S_* (min^−1^)	0.011 ± 0.001	0.011 ± 0.002	0.000 ± 0.000	0.000 ± 0.000	0.007 ± 0.003	0.000 ± 0.000	0.026 ± 0.003	0.152 ± 0.246
	α (-)	0.151 ± 0.038	0.505 ± 0.081	1.000 ± 0.001	1.000 ± 0.007	0.810 ± 0.056	1.000 ± 0.008	0.633 ± 0.036	0.637 ± 1.701
	R^2^_adj_	0.99	0.99	0.99	1.00	1.00	1.00	1.00	1.00
	RMSE	0.04	0.05	0.04	0.02	0.03	0.02	0.02	0.02

## Data Availability

The data presented in this study is available on request from the corresponding author.

## References

[1] Soto M. (2011). Situación y avances tecnológicos en la producción bananera mundial. Rev. Bras. De Frutic..

[2] Happi Emaga T., Andrianaivo R.H., Wathelet B., Tchango J.T., Paquot M. (2007). Effects of the stage of maturation and varieties on the chemical composition of banana and plantain peels. Food Chem..

[3] Schieber A. (2017). Side streams of plant food processing as a source of valuable compounds: Selected examples. Annu. Rev. Food Sci. Technol..

[4] Vu H.T., Scarlett C.J., Vuong Q.V. (2018). Phenolic compounds within banana peel and their potential uses: A review. J. Funct. Foods.

[5] Yang C.-P., Fujita S., Kohno K., Kusubayashi A., Ashrafuzzaman M., Hayashi N. (2001). Partial purification and characterization of polyphenol oxidase from banana (*musa sapientum* L.) peel. J. Agric. Food Chem..

[6] Zaini N.A.M., Osman A., Hamid A.A., Ebrahimpour A., Saari N. (2013). Purification and characterization of membrane-bound polyphenoloxidase (mppo) from snake fruit [*salacca zalacca* (gaertn.) voss]. Food Chem..

[7] Kumar V.B.A., Mohan T.C.K., Murugan K. (2008). Purification and kinetic characterization of polyphenol oxidase from barbados cherry (*Malpighia glabra* L.). Food Chem..

[8] Marques Silva F.V., Sulaiman A., Melton L., Shahidi F., Varelis P. (2019). Polyphenoloxidase in fruit and vegetables: Inactivation by thermal and non-thermal processes. Encyclopedia of Food Chemistry.

[9] Terefe N.S., Delon A., Buckow R., Versteeg C. (2015). Blueberry polyphenol oxidase: Characterization and the kinetics of thermal and high pressure activation and inactivation. Food Chem..

[10] Surowsky B., Schlüter O., Knorr D. (2015). Interactions of non-thermal atmospheric pressure plasma with solid and liquid food systems: A review. Food Eng. Rev..

[11] Kramer B., Hasse D., Guist S., Schmitt-John T., Muranyi P. (2020). Inactivation of bacterial endospores on surfaces by plasma processed air. J. Appl. Microbiol..

[12] Chutia H., Kalita D., Mahanta C.L., Ojah N., Choudhury A.J. (2019). Kinetics of inactivation of peroxidase and polyphenol oxidase in tender coconut water by dielectric barrier discharge plasma. LWT.

[13] Tappi S., Berardinelli A., Ragni L., Dalla Rosa M., Guarnieri A., Rocculi P. (2014). Atmospheric gas plasma treatment of fresh-cut apples. Innov. Food Sci. Emerg. Technol..

[14] Bußler S., Ehlbeck J., Schlüter O.K. (2017). Pre-drying treatment of plant related tissues using plasma processed air: Impact on enzyme activity and quality attributes of cut apple and potato. Innov. Food Sci. Emerg. Technol..

[15] Ünal M.Ü. (2007). Properties of polyphenol oxidase from anamur banana (*Musa cavendishii*). Food Chem..

[16] Chaisakdanugull C., Theerakulkait C. (2009). Partial purification and characterisation of banana [*Musa* (aaa group) ‘Gros Michel’] polyphenol oxidase. Int. J. Food Sci. Technol..

[17] Yang C.-P., Fujita S., Ashrafuzzaman M., Nakamura N., Hayashi N. (2000). Purification and characterization of polyphenol oxidase from banana (*Musa sapientum* L.) pulp. J. Agric. Food Chem..

[18] Galeazzi M.A.M., Sgarbieri V.C. (1981). Substrate specificity and inhibition of polyphenoloxidase (PPO) from a dwarf variety of banana (*Musa cavendishii*, L.). J. Food Sci..

[19] Galeazzi M.A.M., Sgarbieri V.C., Constantinides S.M. (1981). Isolation, purification and physicochemical characterization of polyphenoloxidases (PPO) from a dwarf variety of banana (*Musa cavendishii*, L.). J. Food Sci..

[20] Ngalani J., Signoret A., Crouzet J. (1993). Partial purification and properties of plantain polyphenol oxidase. Food Chem..

[21] Padrón M.P., Lozano J.A., González A.G. (1975). Properties of o-diphenol: O_2_ oxidoreductase from *Musa cavendishii*. Phytochemistry.

[22] Montgomery M.W., Sgarbieri V.C. (1975). Isoenzymes of banana polyphenol oxidase. Phytochemistry.

[23] Palmer J.K. (1963). Banana polyphenoloxidase. Preparation and properties. Plant Physiol..

[24] Gómez-López V.M. (2002). Some biochemical properties of polyphenol oxidase from two varieties of avocado. Food Chem..

[25] Rocha A.M.C.N., Morais A.M.M.B. (2001). Characterization of polyphenoloxidase (PPO) extracted from ‘Jonagored’ apple. Food Control.

[26] Palma-Orozco G., Marrufo-Hernández N.A., Sampedro J.G., Nájera H. (2014). Purification and partial biochemical characterization of polyphenol oxidase from mango (*Mangifera indica* cv. *Manila*). J. Agric. Food Chem..

[27] Mishra B.B., Gautam S., Sharma A. (2012). Purification and characterisation of polyphenol oxidase (PPO) from eggplant (*Solanum melongena*). Food Chem..

[28] Zhou L., Liu W., Terefe N.S. (2018). The inactivation kinetics of soluble and membrane-bound polyphenol oxidase in pear during thermal and high-pressure processing. Food Bioprocess Technol..

[29] Bradford M.M. (1976). A rapid and sensitive method for the quantitation of microgram quantities of protein utilizing the principle of protein-dye binding. Anal. Biochem..

[30] Schweiggert U., Schieber A., Carle R. (2005). Inactivation of peroxidase, polyphenoloxidase, and lipoxygenase in paprika and chili powder after immediate thermal treatment of the plant material. Innov. Food Sci. Emerg. Technol..

[31] Tan T.-C., Cheng L.-H., Bhat R., Rusul G., Easa A.M. (2014). Composition, physicochemical properties and thermal inactivation kinetics of polyphenol oxidase and peroxidase from coconut (*Cocos nucifera*) water obtained from immature, mature and overly-mature coconut. Food Chem..

[32] Goyeneche R., Di Scala K., Roura S. (2013). Biochemical characterization and thermal inactivation of polyphenol oxidase from radish (*Raphanus sativus* var. *Sativus*). LWT Food Sci. Technol..

[33] Illera A.E., Chaple S., Sanz M.T., Ng S., Lu P., Jones J., Carey E., Bourke P. (2019). Effect of cold plasma on polyphenol oxidase inactivation in cloudy apple juice and on the quality parameters of the juice during storage. Food Chem..

[34] Jaramillo Sánchez G.M., Garcia Loredo A.B., Contigiani E.V., Gómez P.L., Alzamora S.M. (2018). Inactivation kinetics of peroxidase and polyphenol oxidase in peach juice treated with gaseous ozone. Int. J. Food Sci. Technol..

[35] Sulaiman A., Soo M.J., Yoon M.M., Farid M., Silva F.V. (2015). Modeling the polyphenoloxidase inactivation kinetics in pear, apple and strawberry purees after high pressure processing. J. Food Eng..

[36] Loomis W.D., Battaile J. (1966). Plant phenolic compounds and the isolation of plant enzymes. Phytochemistry.

[37] Wuyts N., De Waele D., Swennen R. (2006). Extraction and partial characterization of polyphenol oxidase from banana (*Musa acuminata* Grande Naine) roots. Plant Physiol. Biochem..

[38] Griffiths L.A. (1959). Detection and identification of the polyphenoloxidase substrate of the banana. Nature.

[39] Rzepecki L.M., Waite J.H. (1989). A chromogenic assay for catecholoxidases based on the addition of L-proline to quinones. Anal. Biochem..

[40] Terefe N.S., Delon A., Versteeg C. (2017). Thermal and high pressure inactivation kinetics of blueberry peroxidase. Food Chem..

[41] Han Q.-Y., Liu F., Li M., Wang K.-L., Ni Y.-Y. (2019). Comparison of biochemical properties of membrane-bound and soluble polyphenol oxidase from granny smith apple (*Malus* × *domestica* Borkh.). Food Chem..

[42] Surowsky B., Fischer A., Schlueter O., Knorr D. (2013). Cold plasma effects on enzyme activity in a model food system. Innov. Food Sci. Emerg. Technol..

